# Improving the Quality of Patient Care and Healthcare Staff Well-Being through an Empathy Immersion Educational Programme in New Zealand: Protocol of a Feasibility and Pilot Study

**DOI:** 10.3390/mps4040089

**Published:** 2021-12-16

**Authors:** Caz Hales, Chris K. Deak, Tosin Popoola, Deborah L. Harris, Helen Rook

**Affiliations:** School of Nursing, Midwifery and Health Practice, Victoria University of Wellington, Wellington 6012, New Zealand; tosin.popoola@vuw.ac.nz (T.P.); deborah.harris@vuw.ac.nz (D.L.H.); helen.rook@vuw.ac.nz (H.R.)

**Keywords:** empathy, education programmes, feasibility studies, organisational culture, consumer satisfaction, patient satisfaction

## Abstract

Empathy is positively related to healthcare workers and patients’ wellbeing. There is, however, limited research on the effects of empathy education delivered in acute clinical settings and its impact on healthcare consumers. This research tests the feasibility and the potential efficacy outcomes of an immersive education programme developed by the research team in collaboration with clinical partners and a multidisciplinary advisory group. Healthcare worker participants in the intervention ward will receive an 8-week immersive empathy education. The primary outcome (feasibility) will be assessed by evaluating the acceptability of the intervention and the estimated resources. The secondary outcome (efficacy) will be assessed using a quasi-experimental study design. Non-parametric tests will be used to test healthcare worker participants’ empathy, burnout, and organisational satisfaction (within-group and across groups), and healthcare consumer participants’ satisfaction (between-group) over time. Despite growing interest in the importance of empathy in professional relationships, to our knowledge, the present pilot study is the first to explore the feasibility and efficacy of an immersive empathy education in New Zealand. Our findings will provide critical evidence to support the development of a randomised cluster trial and potentially provide preliminary evidence for the effectiveness of this type of empathy education.

## 1. Introduction

### 1.1. Background and Rationale

Empathy is a fundamental component of therapeutic relationships in healthcare, conferring benefits for both healthcare workers (HWs) and healthcare consumers (HCs). For example, empathy is associated with increased wellbeing among healthcare providers, such as greater mental health [1], lower levels of burnout [2], and higher levels of collaboration [3]. Empathy is also related to patients’ psychological wellbeing and physiological outcomes [4]. For example, HWs’ empathy is associated with patients’ tendency to disclose more information [5,6,7], and to better control their chronic health condition [8]. The systematic review of Derksen et al. [4] found that in the presence of physician empathy, patients experienced decreased anxiety and distress and clinical outcomes were enhanced. This perception of physician empathy has even been found to decrease the severity and duration of the common cold [9]. Compassionate and empathetic care factors in patients’ evaluations of their HW [10,11] as shown in patients’ tendency to make personal recommendations for specific physicians [12]. In summary, empathy in healthcare can help HWs to positively influence HCs’ healthcare outcomes as well as contributing to their own feelings of wellbeing.

Despite the benefits of empathy in the clinical setting, evidence suggests that empathy is often missing across the healthcare system [10,13,14,15]. Multiple studies have found that HWs’ empathy declines over the course of medical training [16,17,18,19]. For example, 61% and 23% of surveyed medical residents reported becoming more cynical and less humanistic, respectively, over the course of their residency training [20]. Lack of empathy is associated with higher levels of self-perceived medical error [21], resulting in increased risk to patients. Thus, evidence indicates that there is an emerging need to foster empathy in healthcare.

An individual’s capacity for empathy is influenced by personal and environmental characteristics and can be enhanced through education [22]. Meta-analysis indicated that empathy training sessions and programmes are effective tools to enhance empathy, especially among healthcare professionals and university students [23]. For example, empathy training improved physicians’ ability to read emotional expressions, and resulted in higher empathy scores rated by patients [24]. Furthermore, greater effects were found in studies where participants were compensated for their involvement, empathy measures exclusively focused on understanding and reporting emotions, and when using objective rather than self-reported measures [23]. A systematic review specifically examining empathy education in undergraduate nursing students found that studies that used immersive interventions were the most effective [25], suggesting that immersive education programmes are a promising direction for developing successful education. Despite these advancements in the study of empathy, more research is required to determine the magnitude and duration of effects of different types of empathy training programmes [22,26], different population groups, training settings, and types of assessment measures [23].

In New Zealand, studies on healthcare quality and specifically on empathy are limited. Therefore, measuring national healthcare quality, safety and experience of care has become an overarching national goal set by the Health Quality & Safety Commission [27]. As there is no evidence of exploration of empathy education in the context of the wider healthcare team or its impacts on healthcare consumers in New Zealand, the current research aims to address this gap by developing and testing the feasibility and efficacy of delivering an immersive empathy education programme (EmpEd) to a multidisciplinary healthcare team in the clinical setting. Assessing the feasibility and efficacy of a newly developed empathy education programme is pivotal to develop and design a high quality, high potency education package that effectively supports a healthy ward culture and fosters healthcare consumers’ experiences of empathetic care. This protocol outlines the primary and secondary objectives of this research, the development plan and the implementation of the EmpEd, and the assessment plan of its potential efficacy outcomes.

### 1.2. Objectives

The primary objective of this pilot study is to evaluate the feasibility of the developed immersive Empathy Education Programme in the clinical setting. First, feasibility will be determined by assessing HWs’ acceptance of the intervention. Acceptability will be measured by five indicators: recruitment rates; completion rates; drop-out rates, HW participants’ written feedback; and their oral evaluation of the programme. Second, feasibility will be evaluated by estimating the programme costs and organisational resources needed to facilitate an EmpEd in the future including time commitment, material and financial resources required to run the programme. The secondary objective is to assess the efficacy of the proposed EmpEd. This secondary objective is dependent on the outcome of the feasibility assessment. That is, if the delivered EmpEd’s acceptance level enables us to collect data and ensure acceptable power for statistical analysis, then we will assess HWs’ level of empathy, subjective wellbeing, organisational satisfaction, and HCs’ satisfaction over time.

## 2. Method

### 2.1. Design

This quasi-experimental study of healthcare workers and healthcare consumers is a feasibility and pilot study to form the basis for a future, prospective cluster randomised trial (CRT). The single centre study will be conducted at one tertiary hospital in New Zealand. To minimise harm to participants, the key characteristics that will guide the ward selection are stable workforce, predictable patient population, and stable leadership. Based on local conditions, the Chief Nursing Officer will identify and select two wards (intervention and control ward) that meet these criteria. Although everyone on the wards will be invited to participate in the study, it is reasonable to expect that not everyone would want to be enrolled in the research. Thus, the wards are the study setting, and the groups are the enrolled participants on the wards.

Participants will be pre-allocated into four groups. Based on the participants’ location in the hospital, they will be either part of the intervention or the control group. Based on the participants’ position in the healthcare system, they will be either part of the HW group or the HC group. Participants in the HW groups will be constant during the study, while participants in the HC groups will differ across time points. HWs in the intervention ward/group will receive an 8-week immersive empathy education programme and the control ward/group will operate as usual during the research study. HCs will receive care, support and will interact with healthcare workers as usual in both wards/groups. HCs in the intervention ward/group will not be directly affected by the intervention; however, they will experience limited exposure to the education programme (e.g., seeing educational materials on the ward walls).

**Figure 1:**
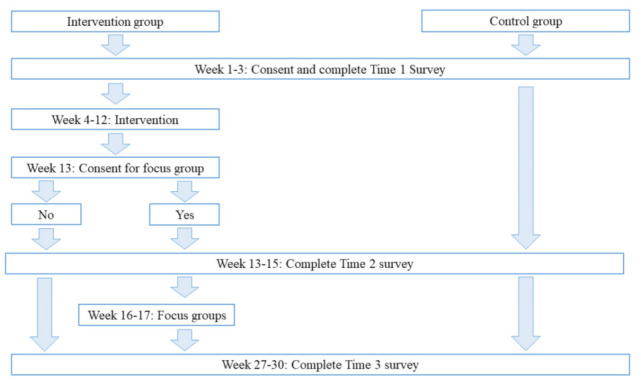
Timetable for participants.

The study duration is expected to be approximately 30 weeks (see Figure 1 for the outline timetable for participants). The study will begin with a 3-week recruitment and consenting period prior to delivering the intervention. Baseline assessment of all participants will be continuous in this 3-week period (Weeks 1–3). This is followed by the 8-week intervention. The research team will deliver the immersion programme in the intervention ward, while the control ward will continue to operate as usual with no interaction/engagement during this time with the research team (Weeks 4–12). After delivering the intervention, all participants will complete the second assessment (Week 13–15). HWs in the intervention ward will be invited to participate in focus group interviews about their experiences of the EmpEd (initiation, Week 13; interviews, Weeks 16–17). Three months later, a final follow-up assessment of all participants will complete the quasi-experiment (Weeks 27–30). The design is adaptive in that the study duration and the intervention can be changed if participation and feedback from study participants indicate necessary adjustments for successful study continuation.

An advisory group will be established, and will be made up of health care professionals, healthcare consumer representatives, cultural advisors, and international experts in empathy education. The purpose of the advisory group is to ensure that relevant perspectives and voices are heard and considered in the design of the intervention and the assessment of the EmpEd (e.g., considering different value orientations in a multicultural environment, the work schedule of HW participants, and access to resources). Three advisory meetings will be held over the course of the programme, and a 100 NZD gift voucher per meeting will be offered to each advisor for their contribution.

### 2.2. Participants

A convenience sample of HW and HC participants will be recruited to complete the study. Participants must provide written, informed consent before any study procedures occur. Eligibility criteria will differ across groups.

HW participants will consist of both registered and non-registered staff, including pharmacists, physiotherapists, occupational therapists, social workers, nurses, doctors, health care assistants, ward clerks, and domestic services staff. With an estimated drop-out rate of 25%, we will recruit up to 138 healthcare worker participants to ensure completion of 110 (55 per ward). Inclusion criteria for healthcare worker participants include (a) being employed in the clinical sites and (b) giving informed consent. Exclusion criteria include (a) working across both the intervention and control wards; (b) agency staff working across multiple wards; or (c) being a medical student, a nursing student, or an allied health student.

HC participants will consist of hospitalised patients and their visiting support persons. With an expected non-completion rate of 10%, we will recruit up to 165 participants to ensure completion of 150 (75 per ward with 25 per timepoint) to complete the study. Inclusion criteria for healthcare consumer participants include (a) ability to understand, read and write English; (b) capacity to give informed consent; (c) (for patients only) being ready for discharge—this is to allow patients to reflect on their experiences and interactions in a non-acute phase of their illness; and (d) (for support persons only) have been actively supporting the patient during their admission (i.e., visited patient at least once per week). Exclusion criteria include (a) being unable to give informed consent; (b) being under the age of 18 years; (c) (for patients only) not predicted to be discharged from the ward between the data collection periods; and (d) (for support persons only) have not been involved in regular support of the patient (i.e., visited less than once a week).

*Risk to participants.* To ensure any risk is minimal in the research, our study design was guided by the following considerations. (1) Appropriate research team member selection: the research team consists of researchers who have expertise in empathy, wellbeing, and coaching. (2) Incorporating self-regulation tools: healthcare worker participants undertaking the EmpEd will be asked to explore differences among people and examine their feelings and attitudes. These activities could raise emotional distress for some participants. To mitigate the risks, the immersion programme has embedded self-care and self-compassion components. (3) Research site selection: the two research sites were selected based on key characteristics—stable workforce, predictable patient population, and stable leadership—to minimise any potential risk to participants. (4) Participant eligibility and exclusion criteria: strict eligibility and exclusion criteria are applied to each potential participant before signing up to the study. (5) Access to workplace Employment Assistance Programme (EAP): if any potential risk should arise that a member of the healthcare team is concerned about, the DHB research team liaison, as well as the principal investigators will be consulted, and appropriate actions will be taken. Non-serious adverse events will be collected systematically during the research and recorded in the case report form.

### 2.3. Procedure

*Recruitment*. To recruit healthcare worker participants, the research team will schedule face-to-face research presentations to nurses and medical professionals on their usual in-service education days. Posters will be placed in the ward areas and research team members will offer information on a one-to-one basis. The nurse managers will also be asked to send out advertisements to their team. Potential participants will be given the participant information sheet and invited to contact a member of the research team if still interested in participating. After the delivery of the intervention, healthcare worker participants from the intervention group will be invited to participate in focus group interviews via email from the research team and verbal invitations from nurse managers. To recruit healthcare consumer participants, advertisements on physical notice boards and table displays will be placed in ward areas. The research team will be present on the wards and invite healthcare consumers on a face-to-face basis to complete the surveys.

*Data collection.* Over the course of the study, quantitative and qualitative data will be gathered at different time points to collect a wide range and type of information for the feasibility and efficacy assessment of the EmpEd. First, surveys will be administered at three time points using Qualtrics software. Each HW participant will be given the survey links via email and will use a unique identifier code, chosen by them, to enter the survey at the commencement of each data collection time point. HC participants will be given a hardcopy to complete with optional assistance provided. Second, two focus groups with approximately 5–10 HW participants from the intervention group will be facilitated after the delivery of the intervention. A research team member, who will not partake in the delivery of the intervention, will organise and facilitate these focus groups to collect qualitative data about the quality and delivery of the programme from the participants’ perspective. The focus group interviews will be transcribed and analysed along with other qualitative data. Finally, during the intervention HW participants will be able to provide hard-copy written feedback about the weekly content of the EmpEd. Participants who discontinue or deviate from the protocol will be excluded from any analysis.

*Participant reimbursement.* To promote participant retention, remuneration for participation will be provided. Payment for each participant is not financially viable, thus financial remuneration is limited to prize draws (i.e., -100 NZD grocery vouchers) for HW participants. Everyone who signed up for the study automatically enters the prize draws. Members of the control group participate in three prize draws at each of the three quantitative data collection periods. The intervention group will enter only one prize draw at the end of the study. Healthcare consumers will receive no reward for participation.

### 2.4. Intervention

There will be two main components to the intervention. A self-directed learning package known as the ‘Aroha Passport’ (Aroha is an indigenous Māori concept that not only incorporates empathy, but also love and compassion and is a deeply emotional way of thinking and being) and researcher-facilitated ward activities.

*Aroha Passport.* The primary aspect of the intervention is facilitating a self-directed learning experience through easily accessible resources. The ‘Aroha Passport’ will be a pocket-sized booklet that consists of eight-weeks of short activities, including reading or writing activities, engaging with work colleagues and short videos relating to a weekly topic (see Appendix A). Each week, the healthcare team will be asked to reflect on a Māori whakataukī (indigenous New Zealand proverb) as well as Western and Eastern ideas to build an understanding of both empathy and how participants relate and respond as people. Participants will be expected to work through the activities and complete the passport in their own time. The time to complete an activity will vary between 2 min and 40 min. Reminders of the activities will be provided via email messages.

*Ward Activities.* The ward will be ‘branded’ with a weekly focus corresponding to the weekly topic of the Aroha Passport. A dedicated staff quiet space for healthcare workers, known as the *Empathy Station*, will be located on the ward. In this space of respite, HW participants will be given a white board activity to reflect on the weekly Māori whakataukī (proverb). These activities include prompts as “What are you going to do this week to take care of yourself?” or “Write a kind word to your patients.” The research team will have an active presence at the empathy station to answer questions and accompany visiting healthcare workers. To demonstrate and role model empathy, a twice daily *hydration station* will be part of the education package. Research team members will walk around with a refreshment trolley, offering different herbal/fruit teas, cold water and occasionally fruits, biscuits, and home baking to working healthcare staff. All other times, the trolley will be located in the empathy station where night staff can access the refreshments. During weekdays, team members will also facilitate a daily 5-min *mindfulness* practice in a designated room with dimmed lighting. These sessions will be either guided by a team member or played from different mindfulness mobile apps to provide a wide variety of experiences to the healthcare staff.

## 3. Measures and Outcomes

### 3.1. Feasibility Outcomes

The feasibility of a prospective CRT on enhancing empathy in the clinical setting will be estimated based on the acceptability of the intervention (1) and the estimated organisational resources needed to continue the programme (2). First, the acceptability of the intervention will be assessed both quantitatively and qualitatively: (a) analysing recruitment, completion, and drop-out rates; (b) monitoring active and passive participation in the intervention; (c) optional weekly written feedback from healthcare worker participants; (d) surveying healthcare worker participants about their prior expectations of the intervention and whether their expectations were met after the intervention; (e) and focus group interviews with approximately 5–10 healthcare worker participants. The main objective of the focus groups is to review and evaluate the EmpEd from the participants’ perspective: evaluation of quality and mode of delivery, usefulness, staff engagement, and specific components of the intervention. Second, to determine the resources needed by the organisation to run an EmpEd, the following need to be considered and estimated: (a) scientific personnel for EmpEd training and supervision; (b) clinical networks and cooperation; and (c) equipment and material costs. Importantly, the EmpEd programme review is not only to measure the feasibility, engagement, and applicability of the programme, but also to assess the viability of continuing aspects of the immersion programme, for example, regular mindfulness sessions or the hydration station.

### 3.2. Efficacy Outcomes

To determine the efficacy of a prospective CRT on enhancing empathy in the clinical setting, we will use quantitative scientific assessment of both HWs and HCs over time. The primary efficacy outcomes will focus on HWs’ empathy levels and their ‘professional wellbeing’ in terms of organisational satisfaction, burnout and work-related positive–negative emotions, which are assessed by three pre-existing scales. Our use of these standardised tools will aid comparability with other studies in subsequent publications.

The *Interpersonal and Social Empathy Index (ISEI*) is a multidimensional measure of empathy, assessing macro perspective taking, cognitive empathy, self–other awareness, and affective response [28]. This 15-item index requires participants to rate the frequency of their experiences about different aspects of empathy on a 5-point scale from 1 (*Never)* to 6 (*Always*). For example, “When I am with someone who gets sad news, I feel sad for a moment too” and “I am good at understanding other people’s emotions”. Items will be averaged to create four distinct scales in which higher scores will indicate higher levels of macro perspective taking, cognitive empathy, self–other awareness, and affective response.

The *Employee Organisation Satisfaction Scale* (*EOSS*) will be used as a proxy to measure the quality of the emotional culture of the clinical unit. This 10-item scale requires participants to indicate their agreement with statements about how they feel towards their organisation (1 = *Strongly disagree*, 5 = *Strongly agree*). For example, “I would recommend my unit as a good place to work” and “At work, my opinion seems to count”. Items will be averaged so higher scores will indicate higher levels of organisational satisfaction as an indicator of healthy emotional ward culture.

The *Professional Quality of Life Scale (ProQOL)* [29] consists of two aspects of one’s feelings about their professional life: a positive (compassion satisfaction) and a negative aspect (compassion fatigue). Compassion satisfaction reflects the pleasure and satisfaction a person derives from their ability to do their work well and to be an effective caregiver. Compassion fatigue is divided into two parts. The first part assesses psychological aspects typical of burnout (e.g., exhaustion, frustration, and depression). The second part focuses on work-related, secondary exposure to extremely stressful events, known as secondary trauma. Each subscale is psychometrically unique and hence does not yield a composite score. Each subscale consists of 10 items, and participants are asked to rate the frequency of their experiences from 1 (*Never)* to 5 (*Very often)*. Items will be averaged together to create three distinct scales. Higher scores will indicate greater work-related satisfaction; a greater risk for job related burnout; and a greater exposure to secondary traumatic stress.

Additionally, at three time points, HW participants will provide answers to open-ended questions about aspects of their professional life related to empathy, for instance, describing their ability to empathetically relate to others in the workplace, sharing the most challenging aspect of expressing empathy towards others and if there is anything that they would like to improve about their ability of relating to others. Qualitative analyses of these written responses will be used to complement the quantitative data on understanding the efficacy outcomes of the EmpEd.

The secondary efficacy outcomes focus on HCs’ experiences of the healthcare team’s empathetic care and support over time. The same survey tools will be administered to HCs who will differ across time points:

The *Consultation and Relational Empathy Measure (CARE)* [30] is a 10-item scale that assesses healthcare consumers’ perception of relational empathy in healthcare workers’ communication. The CARE measure has previously shown good validity and reliability in primary and secondary care [30,31], and has successfully been used to detect change in patients’ perception of resident physicians’ empathy [24]. Instructions will be slightly modified to ask participants to rate the quality of *support* they will have received during their hospital stay/visit (1 = *Poor*, 5 = *Excellent*, with an additional “does not apply” category for each item). Example items include “How good the healthcare team was at making you feel at ease” and “… allowed you to tell your story”. Items will be averaged so that higher scores will indicate higher perceived quality of support.

The *Australian Hospital Patient Experience Question Set (AHPEQS*) will assess patients’ experiences of treatment and care [32]. Participants will rate their level of (dis)agreement with eight statements on a 7-point scale (1 = *Strongly disagree*, 7 = *Strongly agree*, with an additional “does not apply” category). For example, “When a need couldn’t be met, staff explained why”. The AHPEQS measure has previously shown good validity and reliability for patients in hospital care across Australian states [32]. We will trial this measure for use in New Zealand. This trial will support the national assessment of patient experiences in New Zealand undertaken by the Health Quality & Safety Commission [33].

*Measuring HCs’ experiences among visiting support persons.* Both the *CARE* and *AHPEQS* survey tools were designed to assess patients’ experience. There is a lack of validated survey tools assessing satisfaction and quality of care among visiting support persons (e.g., family members of hospitalised patients). Assessing visiting support persons’ views is essential not only for understanding the needs of the wider HC group, but it has pivotal importance in cases where hospitalised patients are unable to participate due to cognitive impairment. For this purpose, the patient experience scales were adapted to measure visiting support persons’ experiences. The survey items remained the same but were reorientated to the perspective of the visitor about their *own* experience of HWs’ treatment of *them* as visitors.

Similarly, qualitative data will be collected in a written format from HC participants about their experience of HWs’ treatment of them on a human level, and whether there is anything more that they wish staff would do for patients and their support persons.

### 3.3. Sample Size Calculation

Two independent HW groups will be assessed across three time points. An estimated 55 staff in each group (110 total) is required to achieve at least 80% power to detect a difference in the outcome measures as a response to the intervention. Applying the Bonferroni correction, the significance level for each test is adjusted to α = 0.05/9 = 0.0056. To assess HCs, initial power calculations suggested that for each HC group, a sample size of 50 per group per timepoint in each ward (300 participants) would result in a confidence interval limit of ±0.139 or 13.9% on a result of 0.5 or 50%. However, we expect that it would be difficult to achieve a sample size of >25 per group. The validity assessment will guide decisions regarding whether combining the two HC groups (patients and support persons) into one HC group per ward per timepoint is advisable. This part of the study is subject to modification and treated as exploratory. Power calculations were conducted in G*Power Version 3.1.9.4 for Windows.

### 3.4. Data Management and Analysis Plan

*Qualitative analysis.* Thematic analysis will be conducted on the feasibility of the EmpEd. The analytic interest is on how the experiences of participants are located within the wider socio-cultural context of the ward, which makes thematic analysis a suitable method for our purpose [34]. Thematic analysis will identify themes addressing feasibility issues, for instance, the usefulness of the programme, staff engagement and sustainability. Interview recordings and observational notes will be transcribed. To ensure consistency and accuracy of the analysis, two members of the research team will jointly analyse the data. The thematic analysis will follow Braun and Clarke’s [35] approach and will be completed by using NVivo 12.

The confidence in the quality of the qualitative data, analysis and interpretation will be assured by following the principles of trustworthiness in qualitative research [36]. Trustworthiness will be achieved by ensuring that multiple researchers have oversight of the data collection and analysis process. To ensure credibility of the data, only HWs who have participated in the EmpEd will be recruited into the focus groups, and verbatim quotes from these participants will be used to support themes/subthemes. The use of NVivo 12 to code the data will enhance opportunities for all researchers to evaluate the process of theme development, and it will also facilitate reflection on the analysis process.

*Quantitative analysis.* Descriptive and inferential statistical analysis will be conducted to assess the feasibility and potential efficacy of the EmpEd. Online responses will be registered through Qualtrics. Hard copy survey responses will be manually entered and error checked by looking at ranges and correlations. A minimum response rate of approximately 75% will be used as a cut-off point for the analysis (i.e., lack of completion of the empathy measures). Descriptive statistics will be summarised by using a number of observations, mean, and standard deviation for continuous data, and frequency and percentage for categorical data. To explore efficacy outcomes, nonparametric analyses will be conducted across time points and between groups due to the potential of a small sample size. Missing data will be reported as missing. All statistical analyses will be performed using R Statistical software.

*Academic Research Output.* The project will produce at least two academic research manuscripts, reflecting the feasibility and efficacy assessment of the EmpEd. These studies will draw comparisons with previous findings on the feasibility of immersive education programmes and their impact in the clinical setting.

*Protocol compliance.* The principal investigators will continuously monitor and overview the research process. Any violations and deviations of the protocol will be addressed on team meetings and recorded in Protocol Violation/Incident reports.

## 4. Discussion

This protocol outlines the process of the feasibility and efficacy assessment of a pilot study on the implementation of an immersive empathy education programme. So far, studying the effects of empathy education has been limited to student samples, and the feasibility of such a programme in clinical settings remains under researched. Assessing the feasibility and acceptability of empathy education is important to establish a programme that is successfully equipped to enhance healthcare workers wellbeing and healthcare consumers’ satisfaction.

Our primary aim is assessing the feasibility of such an intervention because several restraining factors might render the conduct of a CRT in this study setting particularly difficult. For example, reallocation of healthcare workers within the broader hospital unit may result in a high drop-out rate, or the intensive workload may hinder healthcare workers’ engagement and/or motivation to actively participate in the education programme. For this reason, the feasibility assessment of our research includes measures of motivation, workload, intensity of engagement. Furthermore, the study design is adaptive to implement sudden modifications to the intervention during the study. The evaluation of qualitative data collected through written feedback and focus group interviews will further help develop and adapt an empathy education programme which is effective in engaging participants in their daily routine with the training, fostering an empathetic work environment, enhancing healthcare worker participants’ empathy levels and healthcare consumers’ satisfaction.

Our study design is reflective of minimizing several potential risk factors that are detailed above. Here, we address further potential challenges due to the spread of COVID-19 in New Zealand. Country-level COVID-19 imposed restrictions may make it difficult to assess research sites and research participants. Thus, we anticipate that the estimated data collection periods might need to be modified and/or extended in accordance with government guidelines on personal protection and social distancing (e.g., wearing masks, hand sanitizing, and keeping 2 m distance from participants). These potential modifications will allow us to overcome challenges related to the health and safety of the research team and research participants.

The secondary aim of the project is to assess the efficacy of the empathy education programme. This is evidently dependent on the feasibility outcome of the study. If the study provides evidence for its feasibility and acceptability, we may expect that our efficacy findings will generate new knowledge regarding the importance of empathetic healthcare environments for patients and support persons and for those working within the New Zealand health care context. It is anticipated that our findings will highlight the importance of empathy in healthcare and the impact of empathy education on healthcare workers and healthcare consumers in the clinical setting.

The current study has practical implications on how to develop an appropriate research design and foster empathetic workforces to improve the quality of patient care and healthcare workers’ wellbeing. First, assessing the feasibility and efficacy of this pilot study provides essential preliminary insights into the challenges of a future CRT and to develop strategies to successfully implement the programme. For example, understanding what drives motivation and drop-out among HW participants can be used to improve the research design of a CRT. Second, our results will provide practical knowledge and information about the reality of planning and delivering empathy education programmes in healthcare contexts that education providers can use for appropriate curriculum design. The empathy immersion programme, when successful, has the potential to be implemented across all District Health Boards in New Zealand and be adapted for other institutions and organisations who are interested in developing empathetic workforces. Future work is planned to assess the feasibility of the empathy immersion programme across different health care settings. Finally, the insights from healthcare consumers about their satisfaction with healthcare services can contribute to patient safety and lead to a better understanding of healthcare consumers needs, which is in line with the current national efforts to measure patient experience [33].

## 5. Conclusions

The present paper is the protocol of the feasibility and efficacy assessment of an immersive empathy education programme that will be delivered in a tertiary teaching hospital in New Zealand. This research aims to assess the acceptability of the programme and its impact on healthcare workers and healthcare consumers. The results of this project will be disseminated in academic journals and research briefs and will be used to aid the development of future cluster randomised trials.

## Data Availability

The data generated and/or analysed during the current study will not be publicly available due to ethical restrictions. No research data will be kept for longer than 5 years after the conclusion of the research.

## Dissemination Policy

Healthcare worker participants will be invited to attend a research presentation in the clinical setting and have access to a summary report submitted to our clinical partners. Publications will be made available to the healthcare team. An advisory meeting will specifically focus on dissemination strategies to ensure strategic government and non-government organisations have easy accessibility to the findings. We will disseminate our findings to Ministry of Health, Health Quality and Safety Commission, Health and Disability Commissioner, clinical partners, and request publications are included on the *NZ Ministry of Health ‘Health Improvement and Innovation Digest’* website. Dissemination of results will form part of faculty research seminar series and at Grand Round in the District Health Board. Results will also be disseminated in international peer reviewed academic journals and conference presentations.

## Abbreviations

CCDHB	Capital Coast District Health Board
CCSH	Collaborating Centres for Safe Healthcare
CRT	Cluster Randomised Trial
EmpEd	Empathy Education Programme
HC	Healthcare Consumer
HW	Healthcare Worker
HQSC	Health Quality & Safety Commission

## Appendix A. Weekly Topics of the Aroha Passport

- Week 1: Self Compassion—Caring for self
- Week 2: The people you work with—Getting to know those around you
- Week 3: The people who access healthcare—Getting to know patients, family and whānau
- Week 4: Difference—Getting to know your values and the values of others
- Week 5: Kindness—Ways to show kindness towards self and others
- Week 6: Choosing Empathy—Building awareness of your feelings and thoughts about your ability to understand and share in the feelings of others
- Week 7: Listening and noticing—Enhancing communication
- Week 8: Self Compassion Revisited—Caring for self
